# LsrR, the effector of AI-2 quorum sensing, is vital for the H_2_O_2_ stress response in mammary pathogenic *Escherichia coli*

**DOI:** 10.1186/s13567-021-00998-8

**Published:** 2021-10-02

**Authors:** Hui Wang, Fei Shang, Jiawei Shen, Jingyi Xu, Xiaolin Chen, Jingtian Ni, Lumin Yu, Ting Xue

**Affiliations:** 1 School of Life Sciences, Anhui Agricultural University, Hefei, 230036 Anhui China; 2 Institute of Microbe and Host Health, Linyi University, Linyi, 276005 Shandong China

**Keywords:** cow mastitis, mammary pathogenic *Escherichia coli*, H_2_O_2_, AI-2 quorum sensing, LsrR

## Abstract

Mammary pathogenic *Escherichia coli* (MPEC) is an important causative agent of mastitis in dairy cows that results in reduced milk quality and production, and is responsible for severe economic losses in the dairy industry worldwide. Oxidative stress, as an imbalance between reactive oxygen species (ROS) and antioxidants, is a stress factor that is common in most bacterial habitats. The presence of ROS can damage cellular sites, including iron-sulfur clusters, cysteine and methionine protein residues, and DNA, and may cause bacterial cell death. Previous studies have reported that Autoinducer 2 (AI-2) can regulate *E. coli* antibiotic resistance and pathogenicity by mediating the intracellular receptor protein LsrR. This study explored the regulatory mechanism of LsrR on the H_2_O_2_ stress response in MPEC, showing that the transcript levels of *lsrR* significantly decreased under H_2_O_2_ stress conditions. The survival cell count of *lsrR* mutant XW10/pSTV28 was increased about 3080-fold when compared with that of the wild-type WT/pSTV28 in the presence of H_2_O_2_ and overexpression of *lsrR* (XW10/pUClsrR) resulted in a decrease in bacterial survival rates under these conditions. The β-galactosidase reporter assays showed that mutation of *lsrR* led to a remarkable increase in expression of the promoters of *ahpCF*, *katG* and *oxyR*, while *lsrR*-overexpressing significantly reduced the expression of *ahpCF* and *katG*. The electrophoretic mobility shift assays confirmed that LsrR could directly bind to the promoter regions of *ahpCF* and *katG*. These results revealed the important role played by LsrR in the oxidative stress response of MPEC*.*

## Introduction

*Escherichia coli* is one of the main pathogenic bacteria causing clinical mastitis [1, 2]. Mammary pathogenic *E. coli* (MPEC) is a class of extraintestinal pathogenic *E. coli* that usually adheres to and infects dairy cow mammary epithelial cells together with other pathogens, forming biofilms. The biofilm can help pathogens to evade the host immune system and continue to multiply in the mammary gland, leading to persistent intramammary infections [3–5]. As such, research on the role of biofilms in the pathogenesis of mastitis has become important.

Previous studies showed that bacteria express a series of stress proteins to protect themselves from environmental stimuli and to resist adverse environmental pressures such as antibiotics, oxidative stress, acid–base, and osmotic pressure [6–10]. It is generally understood that bacterial infection can trigger innate immune response [11, 12]. Once the pathogenic bacteria infect the host, they are phagocytosed by the host immune cells and macrophages and heterophile cells may produce oxidative bursts in response to pathogens [13, 14]. The rapid production of reactive oxygen species (ROS) including superoxide anions (O_2_^−^), hydrogen peroxide (H_2_O_2_) and the highly reactive hydroxyl radical (·OH) can damage many cellular sites, including iron-sulfur clusters, cysteine and methionine protein residues and DNA, and may eventually cause cell senescence and death [15–17]. H_2_O_2_ is a widely used bactericide for inactivating foodborne pathogens. Over time, a variety of *E. coli* regulatory pathways has evolved to adapt to H_2_O_2_ stress including synthesizing catalases (encoded by *katG* and *katE*), alkyl hydroperoxide reductase (Ahp, encoded by *ahpCF*), and stress protein YciF (encoded by *yciF*). A range of transcriptional regulators including the transcriptional regulator OxyR and the sigma factor RpoS could regulate the synthesis of these enzymes [6, 8, 18–20]. Previous studies showed that *E. coli* resistance to oxidative stress affects both survival and pathogenic potential in the host [21, 22]. However, the detailed molecular mechanism of bacterial adapting to H_2_O_2_ stress needs to be further explored.

Autoinducer 2 (AI-2) is produced by many Gram-negative and Gram-positive bacteria and is considered to be a quorum sensing (QS) signaling molecule involved in interspecies communication [23]. Previous studies have reported that the AI-2 QS system is involved in the bacterial response to oxidative stress [24–26]. The absence of *luxS* (involved in the synthesis of AI-2) in *Streptococcus suis* significantly enhanced its tolerance to H_2_O_2_, and the mutation of *luxS* in *Deinococcus radiodurans* resulted in a significantly higher susceptibility to H_2_O_2_ than wild-type strains [25, 26]. In *E. coli*, the process of AI-2 uptake involves an ATP-binding transporter complex encoding by the *lsrACDB* operon. The expression of the *lsr* operon was regulated by LsrR, a DNA-binding repressor and LsrK, a cognate signal kinase, whose genes (*LsrRK*) are located immediately upstream of the *lsr* operon and divergently transcribed. The LsrR represses the expression of the *lsr* operon and its own *lsrRK* operon by binding to the promoters, while phospho-AI-2 can release LsrR repression and then activating *lsr* operon expression [27–29]. The role of LsrR as a transcriptional regulator mediating QS signal AI-2-related cellular functions has been identified in previous studies and its significant influence on gene expression control has been revealed. According to a previous study, 146 genes were significantly affected by LsrR deletion [30], but whether there is a relationship between LsrR and the regulation of bacterial oxidative stress response has not yet been reported.

In the present study, we demonstrated that the survival ability of *lsrR* mutant under H_2_O_2_ stress was significantly increased compared to that of the wild type. The β-galactosidase reporter assays indicated that LsrR had an obvious inhibitory effect on the expression of *ahpCF, katG,* and *oxyR*. Electrophoretic mobility shift assays (EMSA) confirmed that LsrR inhibited the expression of *ahpcF* and *katG* by direct binding to their promoter regions. This study is therefore helpful to understand the bacterial response to host-derived ROS in MPEC, and might provide potential drug targets for the treatment and prevention of *E. coli* infection.

## Materials and methods

### Bacterial strains, plasmids, and culture conditions

All the strains and plasmids used in this study are listed in Table 1. The *E. coli* strain DCM5 was isolated from milk samples in dairy cows with mastitis and was identified by 16S rDNA sequencing. The *E. coli* cultures were routinely grown at 37 °C in Luria–Bertani (LB) broth (Oxoid, Basingstoke, UK) or on LB agar plates containing 1.5% agar (Oxoid). When needed, antibiotics (Sangon Biotech, Shanghai, China) were used at the following final concentrations: ampicillin (100 μg/mL), chloramphenicol (15 μg/mL), and kanamycin (50 μg/mL).

**Table 1:** **Strains and plasmids used in this study**

Strain or plasmid	Relevant genotype	Reference or source
Strains		
*E. coli*		
DH5α	Clone host strain, supE44 ∆lacU169(ϕ80 lacZ∆M15) hsdR17 recA1 endA1 gyrA96 thi-1 relA1	Invitrogen
BL21	Expression strain, F-ompT hsdS(rB- mB-) gal dcm (DE3)	Invitrogen
WT	*MPEC* (DCM5), wild-type	Laboratory stock
XW10	DCM5 *lsrR*-deletion mutant	This study
WT/pSTV28	WT with the empty vector pSTV28, Cm^r^	This study
XW10/pSTV28	XW10 with the empty vector pSTV28, Cm^r^	This study
XW10/pClsrR	XW10 with the complement plasmid pClsrR, Cm^r^	This study
WT/pUC19	WT with the empty vector pUC19, Amp^r^	This study
WT/pUClsrR	WT with the overexpression plasmid pUClsrR, Amp^r^	This study
WTΔlacZ	DCM5 lacZ-deletion mutant	This study
XW10ΔlacZ	DCM5 lacZ and lsrR mutant strain	This study
WTΔlacZ/pRCL-p_ahpCF_	WTΔlacZ with plasmid pRCL-p_ahpCF_, Cm^r^	This study
XW10ΔlacZ/pRCL-p_ahpCF_	XW10ΔlacZ with plasmid pRCL-p_ahpCF_, Cm^r^	This study
WTΔlacZ/pRCL-p_oxyR_	WTΔlacZ with plasmid pRCL-p_oxyR_, Cm^r^	This study
XW10ΔlacZ/pRCL-p_oxyR_	XW10ΔlacZ with plasmid pRCL-p_oxyR_, Cm^r^	This study
WTΔlacZ/pRCL-p_katG_	WTΔlacZ with plasmid pRCL-p_katG_, Cm^r^	This study
XW10ΔlacZ/pRCL-p_katG_	XW10ΔlacZ with plasmid pRCL-p_katG_, Cm^r^	This study
WTΔlacZ/pRCL-p_katE_	WTΔlacZ with plasmid pRCL-p_katE_, Cm^r^	This study
XW10ΔlacZ/pRCL-p_katE_	XW10ΔlacZ with plasmid pRCL-p_katE_, Cm^r^	This study
WTΔlacZ/pRCL-p_yciF_	WTΔlacZ with plasmid pRCL-p_yciF_, Cm^r^	This study
XW10ΔlacZ/pRCL-p_yciF_	XW10ΔlacZ with plasmid pRCL-p_yciF_, Cm^r^	This study
WTΔlacZ/pRCL-p_rpoS_	WTΔlacZ with plasmid pRCL-p_rpoS_, Cm^r^	This study
XW10ΔlacZ/pRCL-p_rpoS_	XW10ΔlacZ with plasmid pRCL-p_rpoS_, Cm^r^	This study
WTΔlacZ/pRCL-p_katGM6_	WTΔlacZ with plasmid pRCL-p_katGM6_, Cm^r^	This study
WTΔlacZ/pRCL-p_ahpCFM6_	WTΔlacZ with plasmid pRCL-p_ahpCFM6_, Cm^r^	This study
Plasmids		
pKD46	Expresses λ red recombinase Exo, Bet and Gam, temperature sensitive, Amp^r^	[31]
pKD3	cat gene, template plasmid, Amp^r^ Cm^r^	[31]
pCP20	FLP + λcI857 + λpRRep(Ts), temperature sensitive, Amp^r^ Cm^r^	[31]
pSTV28	Low copy number cloning vector, Cm^r^	Takara
pUC19	Cloning vector, Amp^r^	Takara
pUClsrR	pUC19 with lsrR gene, Amp^r^	This study
pClsrR	pSTV28 with lsrR gene, Cmr	This study
pRCL	Cm^r^, promoterless *lacZ*	[46]
pRCL-p_ahpCF_	pRCL harboring *ahpCF* promoter	This study
pRCL-p_oxyR_	pRCL harboring *oxyR* promoter	This study
pRCL-p_katG_	pRCL harboring *katG* promoter	This study
pRCL-p_katE_	pRCL harboring *katE* promoter	This study
pRCL-p_yciF_	pRCL harboring *yciF* promoter	This study
pRCL-p_rpoS_	pRCL harboring *rpoS* promoter	This study
pRCL-p_katGM6_	Mutational pRCL-p_katG_ with 6-bp mutation (‘AACAAT’ to ‘GCGCGC’)	This study
pRCL-p_ahpCFM6_	Mutational pRCL-p_ahpCF_ with 6-bp mutation (‘AAAACT’ to ‘GCGCGC’)	This study
pET28a (+)	Expression vector, Kan^r^	Novagen
pET-lsrR	pET28a (+) with *lsrR* gene, Kan^r^	This study

^*a*^Cm^r^ : chloramphenicol-resistant; Amp^r^ : ampicillin-resistant; Kan^r^ : kanamycin-resistant.

### General DNA manipulation

Genomic DNA from *E. coli* DCM5 was prepared according to the instructions of TIANamp Bacteria DNA Kit (TianGen Biotech, Bei Jing, China). Plasmid DNA was extracted using a plasmid extraction kit (Sangon Biotech, Shanghai, China). PCR testing was carried out using Taq or PrimeSTAR^®^ Max DNA Polymerase (Takara Bio Inc., Dalian, China). The PCR products and DNA restriction fragments were purified using a gel purification kit (Sangon Biotech, Shanghai, China). DNA restriction endonuclease (Thermo Fisher Scientific, Waltham, MA, USA) digestion and DNA ligation (Thermo Fisher Scientific) were performed by standard methods. Primer premier 5.0 software was used for sequence analysis and primer design. The primer sequences used are listed in Table 2.

**Table 2:** **Oligonucleotide primers used in this study**

Primer name	Oligonucleotide (5′–3′)
DCM5-lsrR-f	ATCGTCTCGGCCTGACACGTTTGAAAGTGTCGCGATTGCTTGTAGGCTGGAGCTGCTT
DCM5-lsrR-r	TAATATTCACACTGCACGCCGCGTTAAGCTGCCCGATTCCTGAATATCCTCCTTAGTTC
DCM5-lacZ-f	ATGGTAAGCCGCTGGCAAGCGGTGAAGTGCCTCTGGATGTTGTAGGCTGGAGCTGCTT
DCM5-lacZ-r	GACAATGGTTAAATTGAAATTTGCATAAAAATTGCGGCCTCATATGAATATCCTCCTTA
Check-lsrR-f	GTTGTTGCCCTCAATCTCC
Check-lsrR-r	ACTCCGCCTGTCCCACT
check-lacZ-f	GGCGGTGATTTTGGCGATAC
check-lacZ-r	GTAACGTTGGGTGCAAT
CM-f	TGTAGGCTGGAGCTGCTT
CM-r	CATATGAATATCCTCCTTAGTTC
p_katG_-KpnI -f	GGGGTACCGTGAAAATCACACAGTGATC
p_katG_-BamHI-r	CGGGATCCCAATGTGCTCCCCTCTACAG
p_ahpCF_-HindIII-f	CCAAGCTTTAGATCAGGTGATTGCCCTT
p_ahpCF_-BamHI-r	CGGGATCCCTATACTTCCTCCGTGTT
p_oxyR_-HindIII-f	CCAAGCTTTCCGCAAAAGTTCACGTTGG
p_oxyR_-BamHI-r	CGGGATCCTATCCATCCTCCATCGCCAC
p_katE_-HindIII-f	CCAAGCTTTACTGGCTTCACTAAACGCA
p_katE_-BamHI-r	CGGGATCCTGAACTCGTCTCCTTAATTT
p_yciF_-HindIII-f	CCAAGCTTACCGGAACCAGTTCAACACG
P_yciF_-BamHI-r	CGGGATCCAAGGTGGCTCCTACCCGTGA
p_rpoS_-HindIII-f	CCAAGCTTCCTGATTCACCGTTAATTAT
p_rpoS_-BamHI-r	CGGGATCCTTTTGCCAGTGCCCGGGTT
M6-p_katG_-f	TTAACCGCGCGCATGTAAGATCTCAACTATCGCATCCG
M6-p_katG_-r	TTACATGCGCGCGGTTAAAGAGATGTAGATCAAATTGATCT
M6-p_ahpCF_-f	TTGTAAGGTGCGCGCTATCGATTTGATAATGGAAACGCA
M6-p_ahpCF_-r	ATAGCGCGCACCTTACAACCTTCGTAAGACAACTTT
rt-16 s-f	TTTGAGTTCCCGGCC
rt-16 s-r	CGGCCGCAAGGTTAA
rt-lsrR-f	CGGATCGCGTGGTTTT
rt-lsrR-r	TCAACATATGCGCCGC
rt-ahpCF-f	AGGCATTCAAAAACGGCGAA
rt-ahpCF-r	CGTAGTGGTCAGCAACGTCA
rt-katG-f	GGTGTTGAGAAAGCCGCAAG
rt-katG-r	AGACGAGCGCGATAGTTACG
M13-f	TGTAAAACGACGGCCAGT
M13-r	CAGGAAACAGCTATGACC
T7-f	TAATACGACTCACTATAGGG
T7-r	TGCTAGTTATTGCTCAGCGG
lsrR-EcoRI-f	CCGGAATTCCGTTCAGTTTTGCAGGTGAG
lsrR-KpnI-r	CGGGGTACCTTAACTACGTAAAATCGCCG
lsrR-KpnI-f	GGGGTACCATGACAATCAACGATTCGGT
lsrR-EcoRI-r	CGGAATTCTTAACTACGTAAAATCGCCG
lsrR-NcoI-f	CATGCCATGGACAATCAACGATTCGGT
lsrR-XhoI-r	CCGCTCGAGACTACGTAAAATCGCCG
p-lsrR-biotin-f	ATTTCCCCCGTTCAGTTTTG
p-lsrR-r	AATTCATTCTTCACTTTGAA
p-oxyR-biotin-f	TCCGCAAAAGTTCACGTTGG
p-oxyR-r	TATCCATCCTCCATCGCCAC
p-ahpCF-biotin-f	TAGATCAGGTGATTGCCCTT
p-ahpCF-r	CTATACTTCCTCCGTGTT
p-katG-biotin-f	GTGAAAATCACACAGTGATC
p-katG-r	CAATGTGCTCCCCTCTACAG

^a^The underlined sequences refer to the restriction endonuclease recognition sites.

### Construction of the *lsrR*-deficient mutant

Homologous recombination was used for deletion of *lsrR* from the chromosome of DCM5 based on λ red recombinase system [31]. Briefly, using pKD3 plasmid as a template, the fragment containing the chloramphenicol-resistance cassette (cat) inserted within the *lsrR* gene was amplified with primers DCM5-lsrR-f and DCM5-lsrR-r and then transformed into DCM5 competent cells containing lambda Red recombinase expressed by pKD46 plasmid using electroporation. After electroporation, the cells were added immediately to 900 µL super optimal broth with catabolite repression broth (SOC) without antibiotics, incubated at 37 °C for 60 min, and then mutants were selected by plating cultures on LB plates with 15 μg/mL chloramphenicol. Following incubation for 24 h, the *lsrR* mutant was selected by PCR amplification using the primers check-lsrR-f and check-lsrR-r. Plasmid pCP20 was subsequently transformed into the mutant strain to delete the Cm cassette. The Cm-sensitive mutant strain was confirmed by PCR amplification using the primers check-lsrR-f and check-lsrR-r, and the PCR products were further confirmed by DNA sequencing, with the mutant strain was designated as DCM5ΔlsrR (XW10).

### Complementation of the *lsrR* mutant

To construct the complemented plasmid pSTV28-lsrR, the *lsrR* gene and its promoter region were amplified from the DCM5 strain using the primers lsrR-KpnI-f and lsrR-BamHI-r, and then the PCR products were gel-purified and ligated into the BamHI and KpnI restriction sites of the plasmid pSTV28. The recombinant plasmid pSTV28-lsrR was extracted and further confirmed by PCR using primers M13-f and M13-r, and DNA sequencing. To obtain the complemented strain XW10/pClsrR, the plasmid pSTV28-lsrR was electroporated into the mutant strain XW10. The wild-type strain (WT) and the mutant strain XW10 were transformed with the empty vector pSTV28 to obtain WT/pSTV28 and XW10/pSTV28, respectively.

### Construction of the *lsrR* overexpressing strain

The strain overexpressing *lsrR* was constructed as previously described [32]. The *lsrR* gene was amplified from the DCM5 strain using the primers lsrR-KpnI-f and lsrR-EcoRI-r, and then the PCR products were gel-purified and ligated into the KpnI and EcoRI restriction sites of the plasmid pUC19 to construct the overexpressing plasmid pUC19-lsrR. Subsequently, the recombinant plasmid pUC19-lsrR was extracted and further confirmed by PCR using primers M13-f and M13-r, and DNA sequencing. The purified recombinant plasmid pUC19-lsrR and the control empty vector pUC19 were electroporated into the wild-type strain WT to obtain strains WT/pUClsrR and WT/pUC19, respectively.

### Bacterial growth curves

All strains were cultured overnight in fresh LB broth. Then each sample was diluted to an OD_600_ of approximately 0.03 in 100 mL of fresh LB broth with 15 μg/mL chloramphenicol or 100 μg/mL ampicillin and grown at 37 °C for 28 h with shaking. The OD_600_ was detected at 2 h intervals using a UV/Vis spectrophotometer (Thermo Scientific, Pittsburgh, PA, USA), and the growth curves of these strains were established. The experiment was repeated three times and all samples were measured in triplicate.

### ***H***_***2***_***O***_***2***_*** stress assays***

The overnight cultures were diluted to an OD_600_ of approximately 0.03 in 3 mL of fresh LB broth with 15 μg/mL chloramphenicol or 100 μg/mL ampicillin, and incubated in culture tubes at 37 °C for 2 h with shaking. Then, 6 μL of 30% H_2_O_2_ was added in each experimental group cultures, and the cells continued to be incubated at 37 °C for 1 h with shaking. All cultures from the experimental group and control group (without addition of H_2_O_2_) were then diluted by serial ten-fold dilution with LB medium, and 3 μL of each dilution was dropped onto the LB agar plates and cultured at 37 °C overnight. At the same time, to perform the viable colony-forming units (CFUs) assays, 100 μL of each dilution was spread onto the LB agar plates and cultured at 37 °C overnight. The colonies on each plate were counted. The results were compared between the test groups and the control groups and all experiments were repeated three times.

### β-galactosidase assays

The promoters of *oxyR, katG*, *katE*, *rpoS*, *yciF*, and *ahpCF* were amplified and cloned into the promoterless *lacZ* plasmid pRCL by restriction enzyme digestion and DNA ligation to construct the transcriptional *lacZ* fusion reporter plasmid. To avoid the *lacZ* gene on the chromosome of the strain from affecting the experimental results, the *lacZ* genes from the WT strain and the *lsrR*-deficient strain were mutated using homologous recombination methods based on λ red recombinase system, and then the constructed *lacZ* fusion reporter plasmids were introduced into strain WT/ΔlacZ and XW10/ΔlacZ, respectively. The *E. coli* cells containing the *lacZ* fusion reporter plasmid were cultured in 100 mL LB broth containing a final concentration of 15 μg/mL chloramphenicol for the specified time. After cultivation, cells were collected and diluted in Z-buffer containing 16.1 g/L Na_2_HPO_4_, 5.50 g/L NaH_2_PO4, 0.75 g/L KCl, and 0.246 g/L MgSO4 to 1 mL and assayed for β-galactosidase activity using ortho-Nitrophenyl-β-galactoside (ONPG) as a substrate. The following equation was used to calculate units of enzyme activity: Miller Units: [OD_420_  ×  1000)/(OD_600_  ×  Volume (mL)  ×  Time (min)]. The experiment was repeated three times independently.

### Total RNA isolation, cDNA generation, and real-time reverse transcription-quantitative PCR processing

Real-time reverse transcription-quantitative PCR (RT-qPCR) assays were performed according to a previous study [32]. To detect the transcript levels of *lsrR* in the presence or absence of H_2_O_2_, the overnight cultures of DCM5(WT) cells were diluted at 1:100 and transferred into two parallel fresh 100 mL LB broth. When the cell density reached OD_600_ of 1, the cultures were cultured for another 40 min with or without 50 μL of 30% H_2_O_2_, and then collected for total RNA extraction. To verify the effect of *lsrR* overexpression on the expression of *katG* and *ahpCF*, the overnight cultures of WT/pUC19, and WT/pUClsrR were diluted to an OD_600_ of approximately 0.03 in fresh LB broth with 100 μg/mL ampicillin. The cultures were grown to the exponential phase at 37 °C with shaking. The cells were collected by centrifugation and resuspended in RNase-free water; subsequently, total RNA was extracted from the cells using Trizol reagent [TransGen Biotech (Beijing) Co. Ltd., Beijing, China]. Reverse transcription was carried out using the EasyScript One-Step gDNA Removal and cDNA Synthesis SuperMix kit (TransGen) according to the manufacturer’s instructions. RT-qPCR was performed using the TransStart Tip Green qPCR SuperMix kit (TransGen) with the CFX96™ Real-Time System (Bio-Rad, USA). The 16S cDNA abundance was used to normalize the quantity of the target genes. All the real-time RT-qPCR assays were repeated at least three times.

### Expression and purification of the LsrR protein

The C-terminal His6-tag LsrR protein was cloned and purified according to a previous study [27]. The open reading frame of the *lsrR* gene of *E. coli* DCM5 was amplified with primers lsrR-NcoI-f and lsrR-XhoI-r from DCM5 genomic DNA, and then cloned into the pET28a (+) vector. The recombinant plasmid pET-lsrR was confirmed by PCR using primers T7-f and T7-r, and further confirmed by DNA sequencing. The pET-lsrR vector was then transferred into *E. coli* BL21(DE3) by a chemical transformation method. The LsrR protein was induced to express in *E. coli* BL21(DE3) cells by adding isopropyl β-D-thiogalactopyranoside (IPTG) at a final concentration of 0.5 mM. The purification of His6-tagged LsrR fusion protein was performed with a HisTrap high-performance column as previously described [32]. The purified LsrR protein was stored in 10% glycerol at −80 °C until use. The LsrR protein purity was determined by SDS-PAGE, and the protein concentration was measured by an enhanced BCA protein assay kit (Beyotime, Shanghai, China).

### Electrophoretic mobility shift assay (EMSA)

Four promoter DNA fragments, p-lsrR, p-oxyR, p-ahpCF and p-katG, were amplified from the WT genome by biotin-labeled probe primers (p-lsrR-biotin-f/p-lsrR-r, p-ahpCF-biotin-f/p-ahpCF-r, p-katG-biotin-f/p-katG-r, and p-oxyR-biotin-f/p-oxyR-r) respectively. The biotin-labeled promoter fragments were incubated at 25 °C for 30 min with various amounts of LsrR proteins in 4 µL 5  × binding buffer containing 50 mM Tris–HCl, pH 7.5, 100 mM NaCl, 3 mM magnesium acetate, 0.1 mM EDTA, 0.1 mM dithiothreitol. After incubation, 2 µL 10 × loading buffer with bromophenol blue was added to the mixture and then electrophoresed in a 4% native polyacrylamide gel in a 0.5  ×  Tris–borate EDTA buffer. The band shifts were detected and analyzed according to the manufacturer’s EMSA kit instructions (Beyotime, Shanghai, China).

### Mutation of *ahpCF* and *katG* promoter sites and enzyme activity detection

Mutation of binding sequences of the *ahpCF* and *katG* promoter was performed according to the previous method [27]. There were 6-bp nucleotide bases (AACAAT) in the *katG* promoter region, and 6-bp nucleotide bases (AAAACT) in the *ahpCF* promoter region, which may be necessary for LsrR binding. High ratios of A and T within these motifs also suggest that LsrR might preferentially bind to AT-rich sequences [27] so these 6-bp nucleotide bases in plasmids pRCL-p_ahpCF_ and pRCL-p_katG_ were replaced by the GCGCGC sequence to obtain plasmids pRCL-p_ahpCFM6_ and pRCL-p_katGM6_ (Table 1). The mutations were performed by using Mut Express II Fast Mutagenesis Kit V2 (Vazyme Biotech, Nanjing, China). A DNA fragment about 6.0 kb, containing the full length of the plasmid except the 6-bp nucleotide bases, was amplified by PCR using the plasmid pRCL-p_ahpCF_ or pRCL-p_katG_ as the template and M6-p-ahpCF-f/M6-p-ahpCF-r or M6-p-katG-f/M6-p-katG-r as the primers. The remaining steps were performed according to the Mut Express II Fast Mutagenesis Kit V2 instructions. The constructed mutant plasmid was identified by DNA sequencing then the mutant reporter plasmids were introduced into strain WT/ΔlacZ. The strains WTΔlacZ/pRCL-p_ahpCFM6_ and WTΔlacZ/pRCL-p_katGM6_ were obtained by screening and the expression activity of the mutant promoter was detected by β-galactosidase assays.

### Statistical analyses

Statistical analyses were conducted using the GraphPad Primer 8.0 (GraphPad Software Inc., GraphPad Prism 8.0.1.244, San Diego, CA, USA, 2018) using a one-way ANOVA method. The test results are shown as the mean  ±  SD. A paired t test was used for statistical comparisons between groups. The level of statistical significance was set at a *P *value of  ≤  0.05.

## Results

### Identification of *lsrR* mutant and complementary strains of DCM5

**Figure 1:**
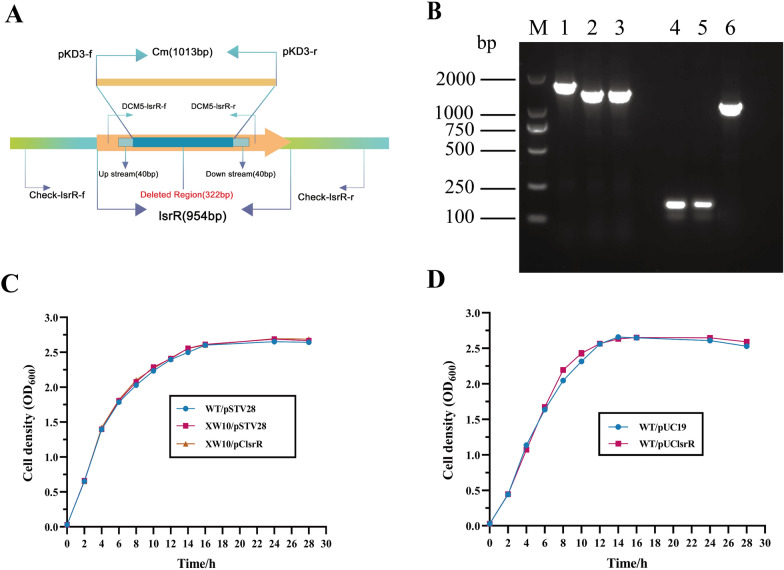
**Molecular determination and growth curve assays of WT, *lsrR* mutant, and complementary strains. A** Schematic diagram of the strategy for constructing the *lsrR* deletion mutant. **B** Confirmation of the wild-type strain WT/pSTV28, mutant strain XW10/pSTV28 and the complementary strain XW10/pClsrR. M: 2000 bp DNA marker; Lane 1: a 1660-bp PCR product was amplified from the strain WT/pSTV28 with primers check-lsrR-f/check-lsrR-r; Lane 2: a 1330-bp PCR product was amplified from the mutant strain XW10/pSTV28 with primers check-lsrR-f/check-lsrR-r; Lane 3: a 1330-bp PCR product was amplified from the complementary strain XW10/pClsrR with primers check-lsrR-f/check-lsrR-r; Lane 4: a 150-bp PCR product was amplified from the strain WT/pSTV28 with primers M13-f/M13-r; Lane 5: a 150-bp PCR product was amplified from the mutant strain XW10/pSTV28 with primers M13-f/M13-r; Lane 6: a 1094-bp PCR product was amplified from the complementary strain XW10/pClsrR with primers M13-f/M13-r. **C** Growth curves of strains WT/pSTV28, XW10/pSTV28, and XW10/pClsrR in LB broth with 15 μg/mL chloramphenicol. **D** Growth curves of strains WT/pUC19 and WT/pUClsrR in LB broth with 100 μg/mL ampicillin. The data represent the means of three independent assays.

The *lsrR* gene was knocked out using homologous recombination to obtain the *lsrR* deletion mutant. The schematic diagram of the strategy for deleting the *lsrR* gene in DCM5 and the primers used for confirmation of the *lsrR* deletion are shown in Figure 1A. The mutant and complementary strains of *lsrR* were confirmed by PCR (Figure 1B). A 1660 bp product was amplified from the wild type strain WT/pSTV28, while 1330 bp products were respectively amplified from the mutant strain XW10/pSTV28 and the complementary strain XW10/pClsrR by using primers check-lsrR-f and check-lsrR-r (Lane 1–3). The plasmid pSTV28 and the complementary plasmid pClsrR were also confirmed by PCR (Figure 1B, Lane 4–6). The PCR products of 100 bp were amplified from strains WT/pSTV28 and XW10/pSTV28 using primers M13-f/M13-r, and a 1094 bp product was amplified from strain XW10/pClsrR.

### *lsrR* deletion and overexpression did not affect strains growth

To ensure that the growth conditions of the mutant XW10 strain and its parent strain were consistent with that of the complementary strain, the empty vector pSTV28 was transformed into the WT and XW10 strains. Growth curve assays were performed and results showed that the growth curves of XW10/pSTV28 and XW10/pClsrR were like that of WT/pSTV28 when cells were cultured in LB broth with 15 μg/mL chloramphenicol (Figure 1C). Likewise, to ensure that the growth condition of the WT strain was consistent with that of the lsrR overexpression strain, the empty vector pUC19 was transformed into the WT strain. The growth curve of strain WT/pUClsrR in LB broth with 100 μg/mL ampicillin was like that of strain WT/pUC19 as shown in Figure 1D.

### Deletion of ***lsrR*** increased bacterial survival ability under the H_2_O_2_ stress

**Figure 2:**
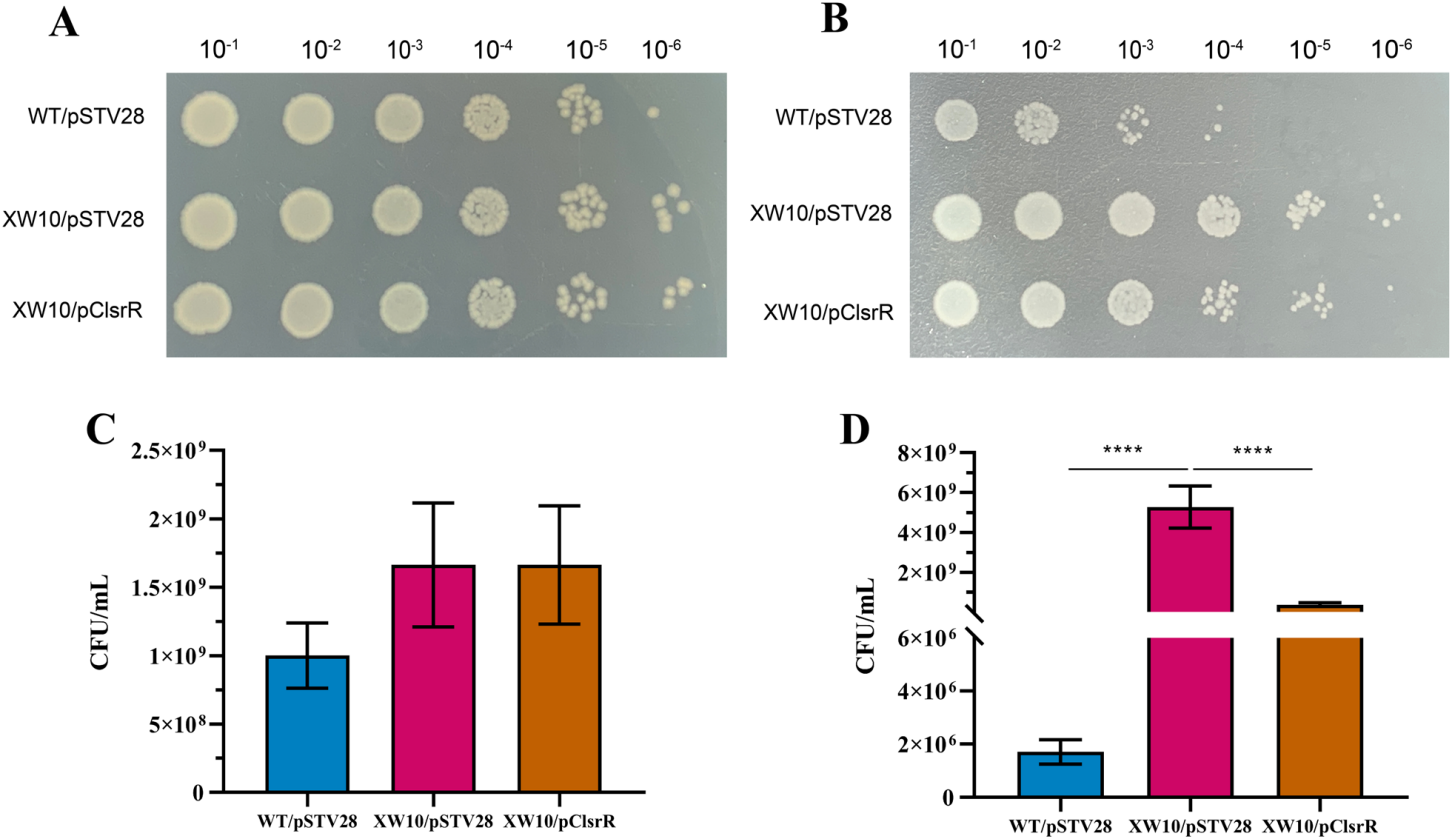
**Analysis of the H**_**2**_**O**_**2**_** stress response of strains WT/pSTV28, XW10/pSTV28, and XW10/pClsrR.** The strains WT/pSTV28, XW10/pSTV28 and XW10/pClsrR with an initial OD_600_ of 0.03 were inoculated into LB broth containing 15 μg/mL chloramphenicol and incubated for 2 h. Then the cultures of the control group (**A**) and the experimental group (treated with 6 μL 30% hydrogen peroxide for 1 h) (**B**) were ten-fold serially diluted, and 3 μL of each dilution was dropped onto LB agar plates and cultured overnight. The above dilutions of the control group (**C**) and the experimental group (**D**) were spread onto the LB agar plates. After incubation overnight, the colony forming units were analyzed. (^∗∗∗∗^*P * <  0.0001).

With the tolerance of pathogenic bacteria to H_2_O_2_ stress being increased, the infecting ability of pathogenic bacteria to the host is also increased. To evaluate the effect of *lsrR* on the survival of strain DCM5 in the presence of H_2_O_2_, the survival ability of the *lsrR*-deficient mutant strain XW10/pSTV28 was compared with that of the wild-type strain WT/pSTV28 and the complementary strain XW10/pClsrR. The results showed that there was no difference in the number of colonies grown on the plates of each group in absence of H_2_O_2_ (Figure 2A). However, the tolerance of strain XW10/pSTV28 to H_2_O_2_ stress was increased when compared with that of strain WT/pSTV28 after treating with H_2_O_2_ for 1 h, and the tolerance was partially restored in strain XW10/pClsrR (Figure 2B). The CFU assays were performed to further analyze the survival rates of strains WT/pSTV28, XW10/pSTV28, and XW10/pClsrR, and the colonies of hydrogen peroxide treatment group and control group on the plates were counted and compared after cultivating in LB broth with 15 μg/mL chloramphenicol overnight. In the absence of H_2_O_2_, there was no difference in the survival rate between the groups as shown in Figure 2C, D. However, with the addition of H_2_O_2_, the survival rate of strain XW10/PSTV28 was increased 3080-fold when compared with that of strain WT/PSTV28, and the survival rate was partially recovered in strain XW10/PClsrR. These results suggested that LsrR played a vital role in the bacterial oxidative stress response.

### Overexpression of ***lsrR*** decreased bacterial survival ability under the H_2_O_2_ stress

**Figure 3:**
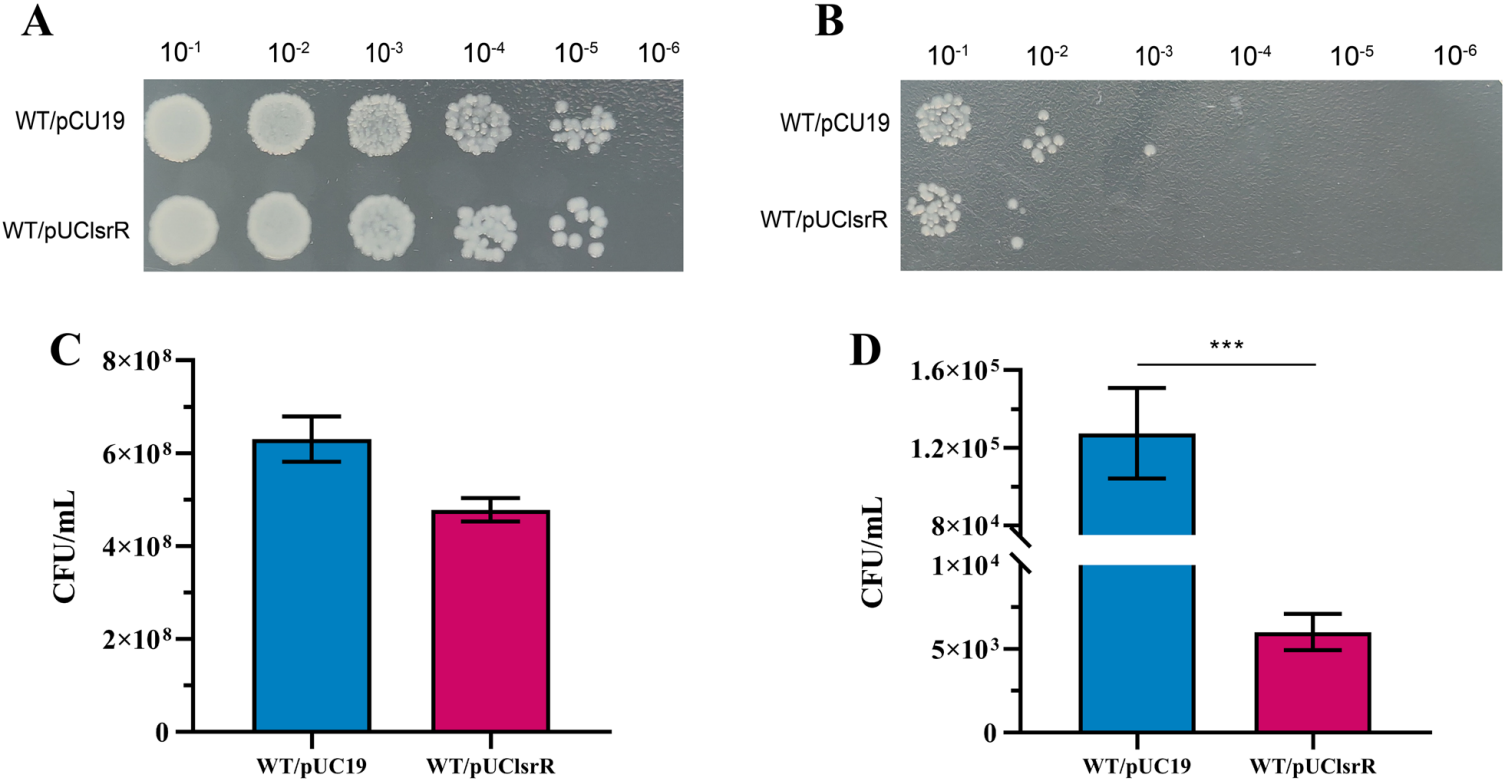
**Analysis of the H**_**2**_**O**_**2**_** stress response of strains WT/pUC19 and WT/pUClsrR. A** The strains WT/pUC19 and WT/pUClsrR with an initial OD_600_ of 0.03 were inoculated into LB broth containing 100 μg/mL ampicillin and incubated for 2 h. Then the cultures of the control group (**A**) and the experimental group (treated with 6 μL 30% hydrogen peroxide for 1 h) (**B**) were ten-fold serially diluted, and 3 μL of each dilution was dropped onto LB agar plates and cultured overnight. The above dilutions of the control group (**C**) and the experimental group (**D**) were spread onto the LB agar plates. After incubation overnight, the colony forming units were analyzed. (^∗∗∗^*P*  <  0.001).

To further evaluate the effect of *lsrR* on the survival of strain DCM5 in the presence of H_2_O_2_, the survival ability of the *lsrR* overexpression strain WT/pUClsrR and its parental strain WT/pUC19 were compared under the H_2_O_2_ stress condition. The results showed that there was no difference in the number of colonies grown on the plates of each group in absence of H_2_O_2_ (Figure 3A), but strain WT/pUClsrR showed reduced tolerance to H_2_O_2_ stress compared with WT/pUC19 after being treated with H_2_O_2_ for 1 h (Figure 3B). To further analyze the survival rates of strains WT/pUC19 and WT/pUClsrR, CFU assays were performed. There was no difference in the survival rate between the groups in the absence of H_2_O_2_ as shown in Figures 3C, D. In the presence of H_2_O_2_, the survival rate of WT/pUClsrR was decreased approximately 21-fold when compared with that of WT/pUC19.This data further supported the important role of LsrR on the oxidative stress response of MPEC.

### Deletion of *lsrR* increased the expression of *oxyR*, *ahpCF*, and *katG*

**Figure 4:**
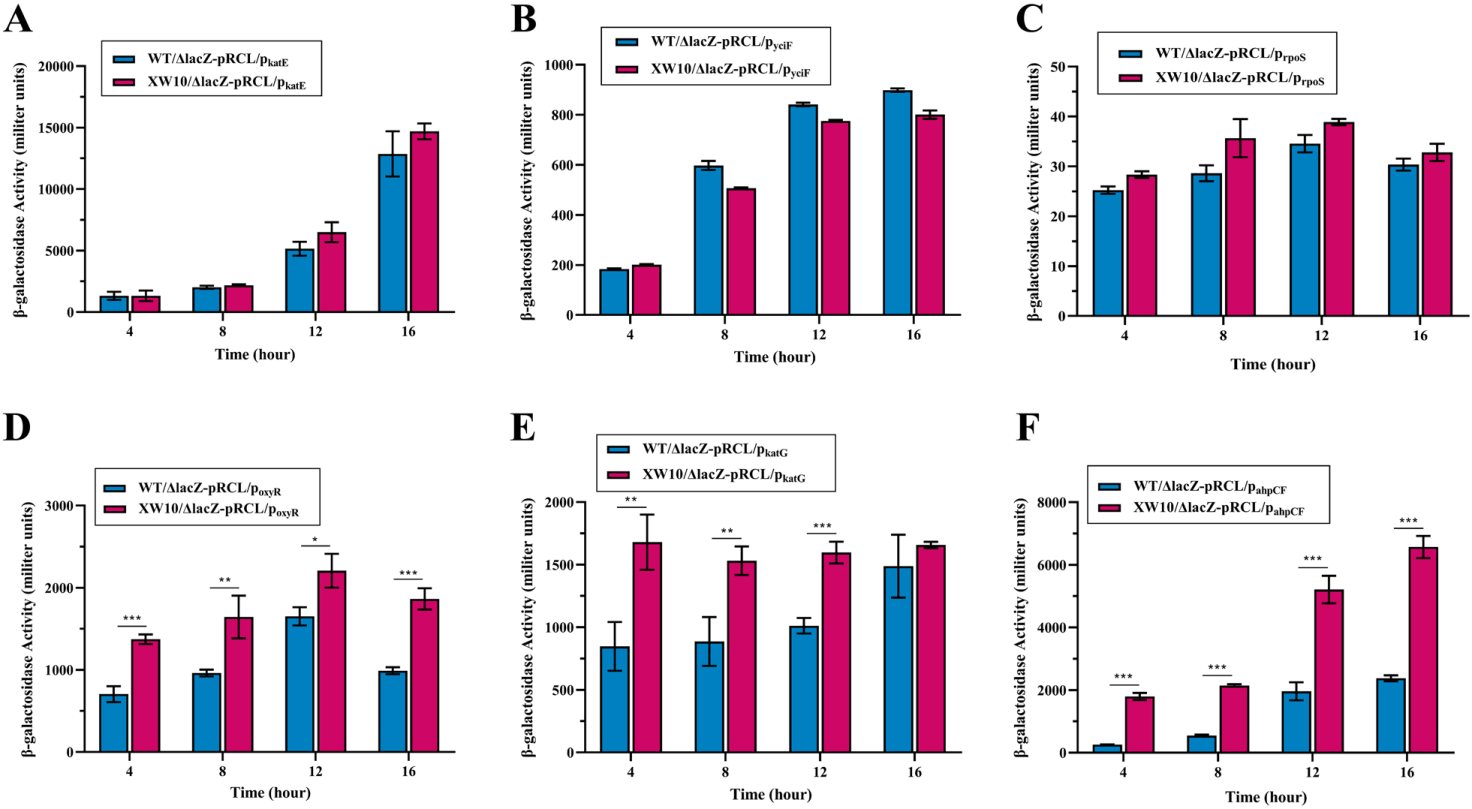
**Measurement of expression activities of the *katE*, *yciF*, *rpoS*, *oxyR*, *katG*, and *ahpCF* promoters.** The β-galactosidase activities expressed in strains WT/ΔlacZ and XW10/ΔlacZ containing plasmid **A** pRCL-p_katE_, **B** pRCL-p_yciF_, **C** pRCL-p_rpoS_, **D** pRCL-p_oxyR_, **E** pRCL-p_katG_ and, **F** pRCL-p_ahpCF_ were measured every 4 h. (^∗^*P * <  0.05, ^∗∗^*P*  <  0.01, ^∗∗∗^*P * <  0.001).

To assess whether the regulatory effects of LsrR on *oxyR*, *rpoS*, *ahpCF*, *katE*, *yciF*, and *katG* were transcriptionally mediated, a system of β-galactosidase report plasmids was constructed to measure expression levels of the promoters of target genes. The data showed that the deletion of *lsrR* had no effect on the transcriptional activities of the *katE*, *yciF*, and *rpoS* promoters (Figures 4A–C). Compared with the WT strain, the deletion of *lsrR* significantly increased the transcription activity of the promoters of *oxyR* (1.95-, 1.71-, and 1.88-fold at 4, 8 and 16 h, respectively), *katG* (1.98-, 1.73-, and 1.58-fold at 4, 8, and 12 h, respectively), and *ahpCF* (7.0-, 3.94-, 2.66- and 2.76-fold at 4, 8, 12 and 16 h, respectively) seen in Figures 4D–F. Taken together, these results suggested that LsrR decreases the bacterial ability to respond to hydrogen peroxide stress by downregulating the expression of hydrogen peroxide stress-associated genes *oxyR*, *katG* and *ahpCF*.

### H_2_O_2_ stress decreased the expression of ***lsrR,*** and overexpression of ***lsrR*** decreased the expression of ***ahpCF***, and ***katG***

**Figure 5:**
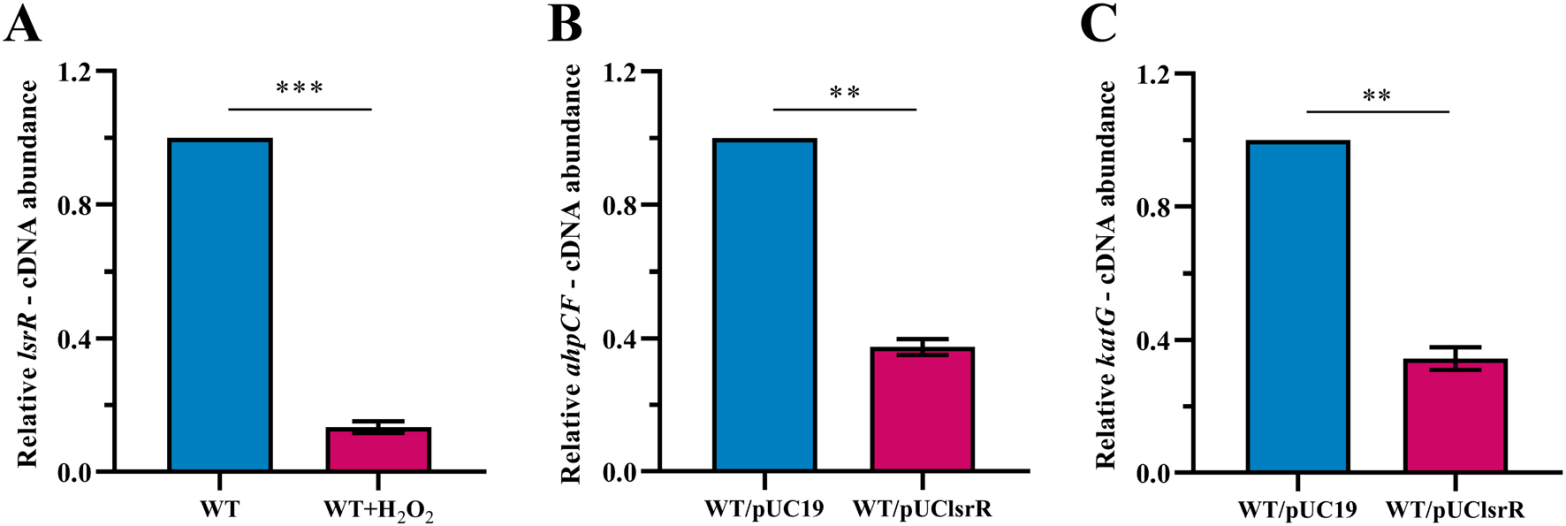
**Comparative measurement of the transcription (complementary DNA [cDNA] abundance) of genes (*lsrR*, *ahpCF*, and *katG*) by RT-qPCR. A** The relative *lsrR* transcript level was determined in WT strain with or without addition 0.5 µL/mL 30% H_2_O_2_. **B** The relative *ahpC* transcript level was determined in strains WT/pUC19 and WT/pUClsrR cultured in LB broth with 100 µg/mL ampicillin. **C** The relative *katG* transcript level was determined in strains WT/pUC19 and WT/pUClsrR cultured in LB broth with 100 µg/mL ampicillin. (Error bars indicate SD; ^∗∗^*P * <  0.01, ^∗∗∗^*P*  <  0.001).

To investigate whether the expression level of *lsrR* in ECM5 was affected by H_2_O_2_ stress, 0.5 μL 30% H_2_O_2_ was added and the transcript level of *lsrR* decreased by 7.5-fold as shown in Figure 5A. The RT-qPCR experiments were performed to determine whether overexpression of *lsrR* affected the transcript levels of the catalase encoding gene *katG* and alkyl hydroperoxide reductase encoding gene *ahpCF*. The data showed that the transcript level of *ahpCF* and *katG* of the *lsrR*-overexpressing strain WT/pUClsrR was decreased by 2.7- and 2.9-fold when compared with its parental strain WT/pUC19 as seen in Figures 5B, C. The expression level of *lsrR* was inhibited by H_2_O_2_ stress and *lsrR* reduced the expression of *ahpCF* and *katG*, so it can be inferred that MPEC can adapt to the H_2_O_2_ stress conditions by mediating LsrR expression.

### LsrR binding to *ahpCF* and *katG* promoters

**Figure 6:**
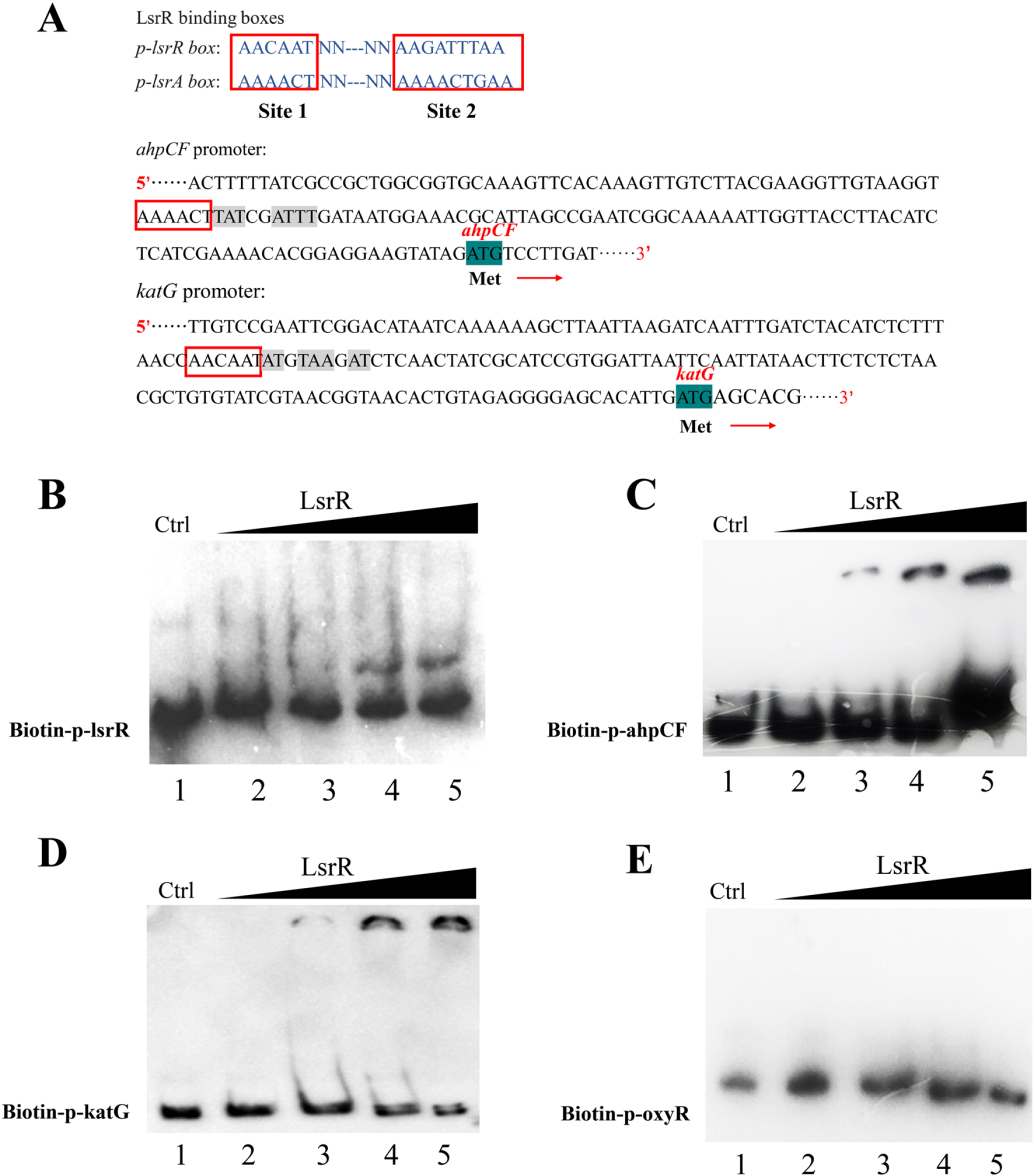
**The binding ability of LsrR to the p-ahpCF-box, p-katG-box, and p-oxyR-box was determined by gel shift assays**. In each panel, from lanes (1) to (5), the LsrR concentration was 8, 0, 2, 4, and 8 mmol, respectively; the amounts of biotin-labeled probes in all lanes were 100 fmol. In lane 1, besides the labeled probes, 1 pmol unlabeled probes were added as the competitive control (Ctrl). **A** Site1 and site2 represent two known binding boxes of LsrR. The red boxes indicate two putative binding sites of LsrR in the promoter regions of *ahpCF* and *katG*, respectively. **B** The binding ability of LsrR to its promoter (positive control). **C** The binding ability of LsrR to the *ahpCF* promoter. **D** The binding ability of LsrR to the *katG* promoter. **E** The binding ability of LsrR to the *oxyR* promoter.

Our previous study showed that *lsrR* and *lsrA* promoter regions have two LsrR binding boxes, AAAACT and AAAACTGAA in the p-lsrA-box and AACAAT and AAGATTTAA in the p-lsrR-box, and the motifs contain high ratios of A and T [27]. On this basis, it was speculated that a putative binding site of LsrR existed in the promoter regions of *ahpCF* and *katG* genes (Figure 6A) and the results suggested that LsrR might repress the two genes by direct binding to their promoter regions. According to sequence alignment, the putative binding site of LsrR in the *oxyR* promoter region was not found.

To verify the above hypothesis, electrophoretic mobility shift assays were performed to confirm whether LsrR binds to the *oxyR*, *ahpCF* and *katG* promoter regions. A positive control assay confirmed that LsrR could bind to its own promoter region (Figure 6B). As shown in Figure 6C, D, clearly shifted bands of protein-DNA complex were detected at LsrR concentrations of 4, 8 and 12 mM, and the intensity of the shifted band was enhanced as the amount of LsrR was increased. The results confirmed that LsrR could specifically bind to the promoter regions of *ahpCF* and *katG*, indicating that LsrR could directly regulate the transcription of *ahpCF* and *katG*. However, the shifted band of the protein-DNA complex was not detected when the promoter region of *oxyR* was used as a probe (Figure 6E), suggesting that LsrR cannot bind to the *oxyR* promoter regions. This suggested that LsrR might regulate the expression of *oxyR* through several indirect pathways and indicated that it had a complicated regulatory pattern in the regulation of bacterial oxidative stress response.

### The β-galactosidase assays of the mutated promoters of *ahpCF* and *katG*

**Figure 7:**
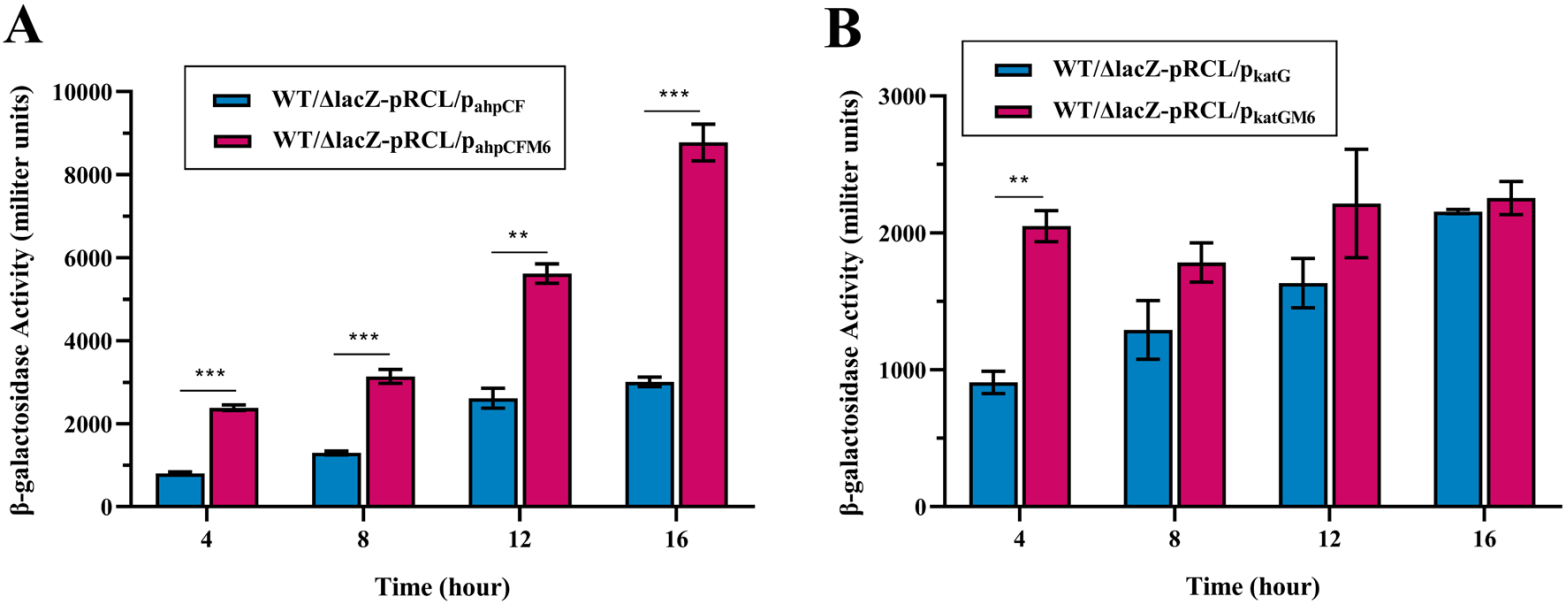
**Expression activities of the *ahpCF* and *katG* promoters and their mutant promoters.** β-galactosidase activities of the strains with plasmids **A** pRCL-p_ahpCF_ or pRCL-p_ahpCFM6_, **B** pRCL-p_katG_ or pRCL-p_katGM6_ were measured every 4 h. (^∗∗^*P*  <  0.01, ^∗∗∗^*P*  <  0.001).

Based on sequence alignment, it was speculated that a putative binding site of LsrR existed in both promoter regions of *ahpCF* and *katG* genes. The EMSA results showed that LsrR could directly bind to the promoter regions of *ahpCF* and *katG*, which led to the hypothesis that these two putative sequences may be crucial for LsrR binding. To further verify this, mutant β-galactosidase reporter plasmids pRCL-p_ahpCFM6_ and pRCL-p_katGM6_ were constructed and the data in Figure 7A showed that compared with pRCL-p_ahpCF_, pRCL-p_ahpCFM6_ was associated with an apparent increase in the expression of *lacZ* throughout the growth cycle. The expression of *lacZ* in pRCL-p_katGM6_ increased only before 4 h compared with pRCL-p_katG_ as shown in Figure 7B, so these results suggested that the sequence AAAACT in the promoter region of *ahpCF* was essential for the binding of LsrR in DCM5 strain. However, mutation of AACAAT in the promoter region of *katG* did not reduce the binding ability of LsrR throughout the growth cycle.

## Discussion

Bacteria can precisely regulate gene expression to promote their survival in various environments or hosts. Bacterial AI-2 quorum sensing is common and is involved in a variety of biological processes, including signal transduction, drug resistance, pathogenicity, motility, and interbacterial competition [23, 33–36]. In *E. coli*, AI-2 can regulate some physiological processes through the AI-2 receptor protein LsrR and the AI-2 metabolic pathway. The previous study showed that AI-2 relies on the intracellular receptor LsrR to increase the β-lactam antibiotic resistance of a broad-spectrum β-lactamase-positive *E. coli* isolated from cows with mastitis [37]. Another subsequent study indicated that exogenous AI-2 can down-regulate the transcription of folate biosynthesis-related genes *folA*, *folC*, *luxS*, *metE*, and *metH* through non-LsrR-dependent pathways, increasing avian pathogenic *Escherichia coli* (APEC) sensitivity to trimethoprime-sulfamethazole [38]. These studies supported the regulatory role of AI-2 or LsrR in bacterial adaption to environmental stimulus. However, whether there is a relationship between LsrR and the regulation of bacterial oxidative stress response has not been reported yet. This study explored the relationship between LsrR and bacterial oxidative stress regulation in MPEC and clarified the regulatory details of LsrR on the oxidative stress-related genes.

Oxidative stress can trigger multiple responses that have evolved as complex regulatory systems in bacterial pathogens, aiding bacteria to cause persistent infections [39–41]. A previous study indicated that the LsrR controls invasiveness of *Salmonella* Typhimurium by regulating *Salmonella* pathogenicity island-1(*SPI-1*) and flagella genes [42]. A subsequent study demonstrated that the *lsr* operon is involved in mediating the virulence of APEC but the regulatory mechanism remains unclear [43]. In *Xanthomonas oryzae* pv. *oryzae*, OxyR degrades hydrogen peroxide in rice by regulating the expression of catalase CatB, and then promotes bacterial infectivity [44]. The data from this study showed that the expression of LsrR was affected by H_2_O_2_ stress in the environment and that LsrR can directly regulate the expression of oxidative stress response associated genes *ahpCF* and *katG* suggesting that MPEC might respond to the H_2_O_2_ stress in an LsrR-dependent pathway. Because the ability to respond to oxidative stress of bacteria is very important for its infection process in the host, it was speculated that LsrR may also be associated with the regulation of bacterial pathogenicity in MPEC, so it was important to explore the regulatory role of LsrR on bacterial pathogenicity or other important physiological processes in future work.

The EMSA results showed that LsrR directly binds to the promoter regions of *ahpCF* and *katG*, which led to the hypothesis that p_ahpCF_: AAAACT and p_katG_: AACAAT may be crucial for LsrR binding. To verify this, mutant β-galactosidase enzyme activity reporter plasmids pRCL-p_ahpCFM6_ and pRCL-p_katGM6_ were constructed and their β-galactosidase activities were measured. The results suggested that the AAAACT sequence in the promoter region of *ahpCF* was essential for the binding of LsrR in DCM5 strain, which was consistent with our previous results [27], but the mutation of the AACAAT sequence in the promoter region of *katG* did not promote the transcriptional activity of *katG* promoter throughout the growth cycle. The previous study also has shown that LsrR binding sequences contained high ratios of A and T, but do not need to share a high level of sequence homology [27]. A subsequent study indicated that mutation of nucleotides in the vicinity of p-lsrR-box sequence AACAAT–AAGATTTAA also affected the transcriptional activity of the *lsrR* promoter, which might be associated with binding of CytR repressor [45], therefore it was speculated that the mutation of the binding site in the *katG* promoter region may cause the recognition and binding by other transcriptional repressors.

**Figure 8:**
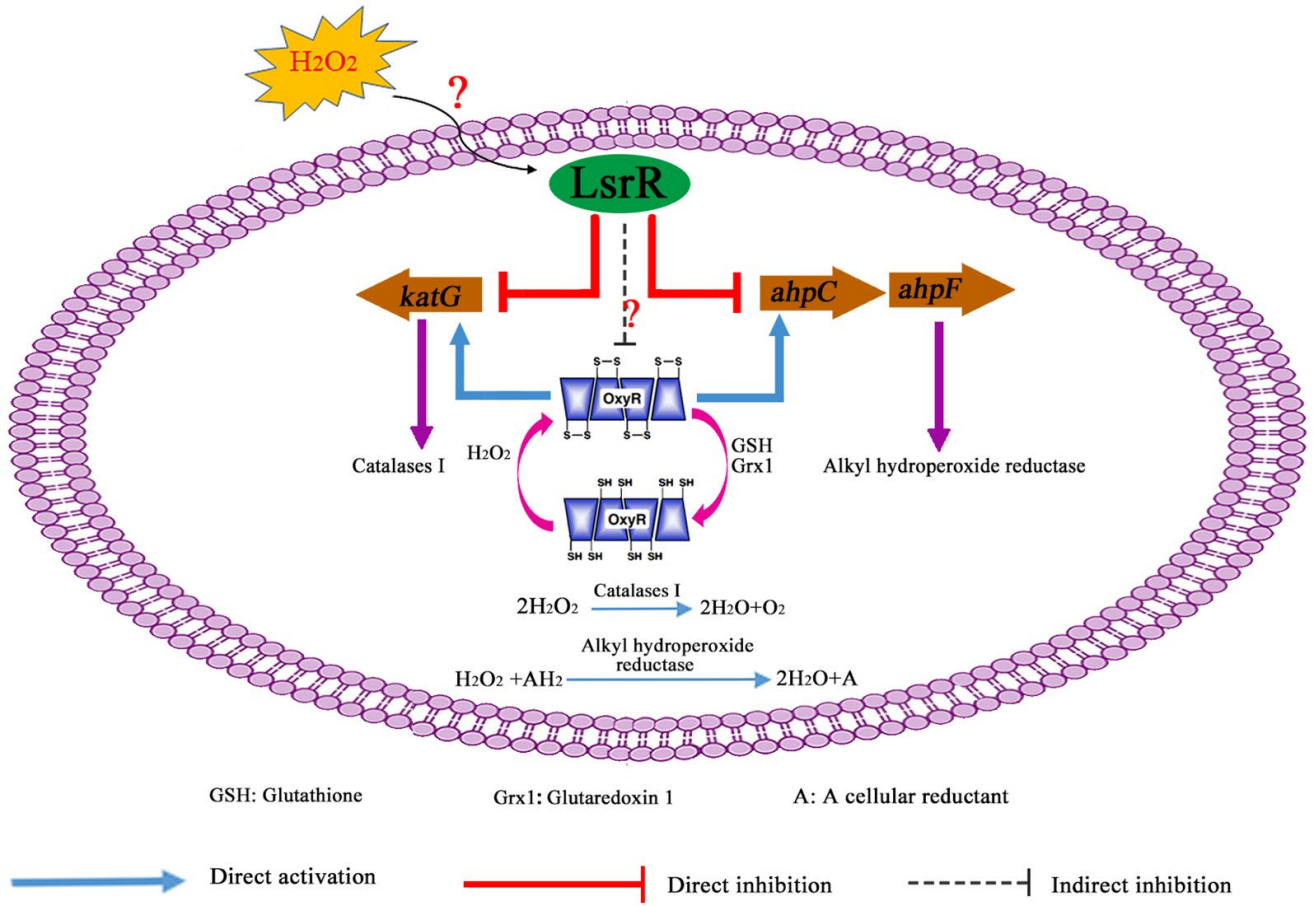
**Schematic diagram of the LsrR-mediated regulation on bacterial H**_**2**_**O**_**2**_** response in MPEC.**

According to present and previous studies, a schematic diagram was made to illustrate the regulatory mode of LsrR on the H_2_O_2_ stress response of MPEC (Figure 8). These data showed that LsrR decreased H_2_O_2_ stress tolerance ability of MPEC by directly or indirectly inhibiting the transcriptions of H_2_O_2_ stress response associated genes including *ahpCF*, *katG*, and *oxyR.* The transcription of *lsrR* exhibited a significant decrease in response to the H_2_O_2_ stress. These results led to the conclusion that MPEC can adapt to H_2_O_2_ stress conditions by reducing the transcription of *lsrR*, which contributed to increased expression of H_2_O_2_ stress response associated genes. But how this bacterium regulates the *lsrR* expression under the H_2_O_2_ stress conditions remains obscure and needs to be further explored.

## Abbreviations

*E. coli*: *Escherichia coli*
MPEC: mammary pathogenic *E. coli*
APEC: avian pathogenic *Escherichia coli*
ROS: reactive oxygen species
H_2_O_2_: hydrogen peroxide
QS: quorum sensing
IPTG: isopropyl β-D-thiogalactopyranoside
LB: Luria–Bertani
AI-2: autoinducer 2
ONPG: ortho-Nitrophenyl-β-galactoside
CFU: colony-forming units
PCR: polymerase chain reaction
RT-qPCR: real-time reverse transcription-quantitative PCR
